# A 37–40 GHz 6-Bits Switched-Filter Phase Shifter Using 150 nm GaN HEMT

**DOI:** 10.3390/nano13202752

**Published:** 2023-10-12

**Authors:** Jae-Hyeok Song, Eun-Gyu Lee, Jae-Eun Lee, Jeong-Taek Son, Joon-Hyung Kim, Min-Seok Baek, Choul-Young Kim

**Affiliations:** Department of Electronics Engineering, Chungnam National University, Daejeon 34134, Republic of Korea; sjh6248@o.cnu.ac.kr (J.-H.S.);

**Keywords:** 5G NR (n260), GaN HEMT, phase shifter, STPS, T-type filter

## Abstract

In this paper, we present a 6-bit phase shifter designed and fabricated using the 150 nm GaN HEMT process. The designed phase shifter operates within the n260 (37~40 GHz) band, as specified in the 5G NR standard, and employs the structure of a switched-filter phase shifter. By serially connecting six single-bit phase shifters, ranging from 180° to 5.625°, the designed phase shifter achieves a phase range of 360°. The fabricated phase shifter exhibits a minimum insertion loss of 5 dB and an RMS phase error of less than 5.36° within the 37 to 40 GHz. This phase shifter is intended for seamless integration with high-power RF circuits.

## 1. Introduction

With the emergence and widespread adoption of the 5th generation communication (5G), the 3rd Generation Partnership Project (3GPP) has established millimeter wave frequency bands, such as n257 (26.5~29.5 GHz) and n260 (37~40 GHz), as standards for 5G New Radio (NR). These defined frequency bands are now being adopted by numerous countries, necessitating the support for mmWave systems to enable cross-network roaming of User Equipment (UE) devices [1,2]. In high-frequency wireless communication systems, phased arrays play a crucial role in effectively concentrating the transmitter output power. It is crucial to provide appropriate phase and amplitude control signals to individual array elements [3]. Phased array systems utilize beam steering techniques to enhance the signal-to-noise ratio (SNR) and achieve spatial selectivity [4].

Phase shifter (PS) is one the most important elements in a phase array system. To facilitate beam steering, it is essential to have phase shifters at each Radio Frequency (RF) front-end. These phase shifters adjust the phase according to the control signal, enabling coverage of up to 360 degrees of phase adjustment. Fluctuations in gain and input/output reflection within different phase states affect channel quality. Moreover, when considering the number of channels, power consumption, and die size, a phase shifter must achieve a balance among phase resolution, die area, and input/output reflection to ensure optimal performance, efficiency, and cost [5].

The GaN (Gallium Nitride) process finds extensive application in high-power, high-frequency scenarios. Gallium Nitride (GaN), known for its higher breakdown strength, faster switching speed, enhanced thermal conductivity, and lower on-resistance, has led to the development of power devices that outperform their silicon-based counterparts [6,7]. These attributes make GaN highly versatile in high-power, high-frequency applications. GaN-based phase shifters are currently in development, aiming for seamless integration into other RF circuits, particularly high-power amplifiers (PAs). These GaN-based phase shifters demonstrate exceptional characteristics, including high-power handling capacity, outstanding efficiency, and robust linearity [8,9,10].

Phase shifters can be categorized into two main types: passive phase shifters [5,11,12,13,14,15,16,17,18] and active phase shifters [19,20,21]. Active phase shifters offer a signal gain advantage, but their gain stage must meet stringent linearity requirements in an RF front-end. While this linearity can be improved by increasing DC power consumption, it comes at the cost of reduced power efficiency. Active phase shifters rely primarily on a vector summing technique, and any phase or amplitude mismatches can significantly degrade their accuracy. Refs. describe vector sum phase shifters, which often exhibit high-power consumption [19,20,21]. On the other hand, passive phase shifters have distinct advantages, including minimal power consumption and exceptional linearity. When integrated at the transmit/receive (TRx) points within a phased array system, their bidirectional capability can reduce the total number of required phase shifters through efficient sharing [22]. However, it is worth noting that passive phase shifters may introduce some insertion loss and often require multiple bits to achieve the desired phase resolution. In conclusion, passive phase shifters are a suitable choice for high-power RF systems where linearity is critical.

The passive phase shifter (PS) can be categorized into several types: transmission line type [16], load-adjusted reflective type [17,18], all-pass network type [11], passive vector sum modulator [5], and switched filter type [12,13,14,15]. Among these topologies, the transmission line type occupies a larger chip area due to considerations of wavelength. In contrast, the load-adjusted reflective type of passive PS boasts a compact design; however, its single-order structure places limits on achievable phase-shifting ranges. The all-pass network (APN) type showcases exceptional bandwidth performance in lower-frequency designs. The passive vector sum modulator (PVSPS) offers superior phase resolution. Nevertheless, its intricate structure and high insertion loss, attributed to the utilization of an X-attenuator, introduce significant drawbacks. Switched filter-type PS (STPS) has no limitations in phase shift range, is suitable for high-frequency applications, and features a simple structure. There is a concern regarding chip area because each phase shifting stage includes inductors. Unlike CMOS, compound processes such as GaN have a higher Q factor due to wider spacing between substrates and lower transmission loss. These characteristics facilitate the design and utilization of inductors [23,24,25]. GaN processes are also suitable for the implementation of switch-filter type phase shifters due to the simplified structural design methods required due to constraints on available metal layers.

In this paper, we present a phase shifter designed for high-power phased array systems operating in the n260 (37~40 GHz) frequency band in line with the 5G NR standard. The designed phase shifter employs a structure and process suitable for high-power systems and consists of a 6-bit STPS (Switched-Type Phase Shifter) configuration. It has been finely tuned to minimize insertion loss and phase error. The phase shifter was fabricated and measured using a 150 nm GaN HEMT process.

## 2. Design of 6-Bits Switched-Type Phase Shifter

The switched-type phase shifter (STPS) operates by controlling multiple single-bit phase shifters (SBPS) using a digital control signal to achieve the desired overall phase adjustment. This involves arranging each SBPS in a series configuration. The STPS is built around a foundational 180° SBPS circuit and requires multiple bits to achieve precise beam steering. However, limitations related to insertion loss and the physical size of each SBPS impact its configuration. Additionally, variations in performance and phase errors result from the phase control and the positioning of individual SBPS units. Consequently, a comprehensive design approach is necessary to consider all possible phase control states.

**Figure 1:**
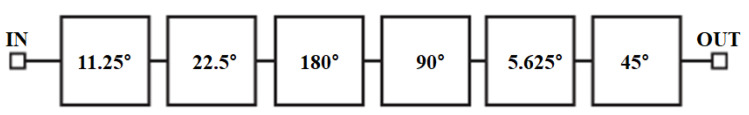
Block diagram of 6-bit phase shifter.

As depicted in Figure 1, the block diagram showcases a 6-bit phase shifter design. By arranging six SBPS units in series, spanning from 180° to 5.625°, a beam steering range of 360° is attainable with a resolution of 5.625°. The phase shifter’s sequence is structured as 11.25°/22.5°/180°/90°/5.625°/45°. This order takes into account phase shifter performance and input/output return loss, and since the characteristics of 90° and 180° SBPS are sensitive, they are located in the center.

A delay element used within the delay path can adopt an LC filter structure comprising T and Pi types. The choice was made to employ the LCL structure, forming a low-pass T-type filter, given its suitability for the relevant frequency band and implementation considerations. GaN HEMT processes offer the advantage of using inductors with high Q values and more than twice the inductance over the same length compared to CMOS processes. Consequently, opting for an LCL-based T-type filter is advantageous. To capture the influence of parasitic elements, electromagnetic (EM) simulations were conducted for the complete layout.

**Figure 2:**
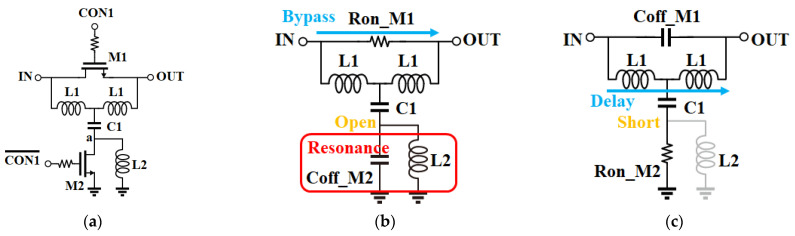
Schematic of (**a**) single-bit phase shifter with low-pass T-type; (**b**) bypass state; (**c**) delay state.

Figure 2 presents a schematic of an SBPS featuring a low-pass T-type filter, along with the corresponding equivalent circuit contingent upon the selected path. Transistor M1 is chosen to have a suitably low on-resistance in the bypass state, while the values of L1 and C1 are established to ensure the desired phase in the delay state, as determined by Equation (1). Transistors M2 and L2 are configured to either open or short node ‘a’ based on the selected state.

(1) $$L1=\frac{Z_{0}\tan\left|\frac{\varphi }{2}\right|}{\omega_{0}},\;C1=\frac{\sin\left|\varphi \right|}{\omega_{0}Z_{0}}$$

All SBPS units, except for the 180° configuration, were designed using a single filter. However, a single filter-based phase shifter is limited to achieve phase delay more than 90° [26]. Therefore, two 90° phase shifters were arranged in series to achieve the 180° SBPS configuration.

**Figure 3:**
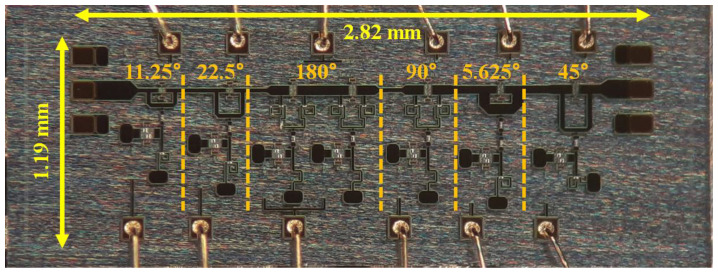
Photomicrograph of fabricated phase shifter.

Figure 3 displays a photomicrograph of a phase shifter designed for the n260 (37–40 GHz) frequency band, manufactured utilizing the 150 nm GaAs HEMT process. The size of the phase shifter is 2.82 × 1.19 mm^2^, encompassing RF and DC pads. For phase shift control, the control voltage was applied through wire bonding to a printed circuit board (PCB). Furthermore, on-wafer measurements were conducted using a Ground-Signal-Ground (GSG) probe tip for RF signal.

The control voltage is set to 0 V when the transistor is active and ranges from −2.5 V to −5 V when it is inactive. The characteristics of the off-state transistor vary depending on the control voltage. The minimum root mean square (RMS) phase error was attained when the control signal operated at −3 V. Since there was not a significant impact on the matching characteristics and insertion loss based on the off-state control voltage, we selected −3 V to minimize the RMS phase error.

## 3. Fabrication and Measurement

**Figure 4:**
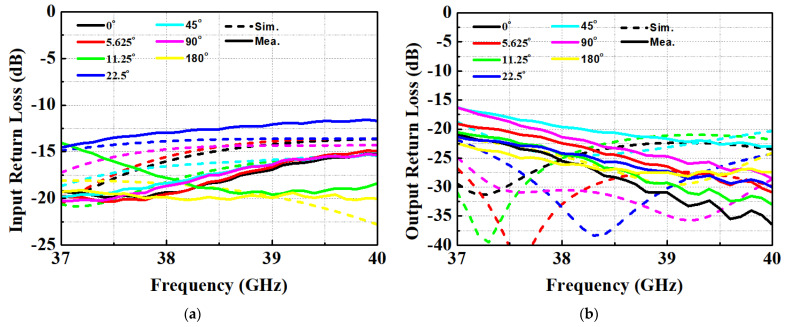
Return loss of n260 phase shifter: (**a**) Input; (**b**) Output.

**Figure 5:**
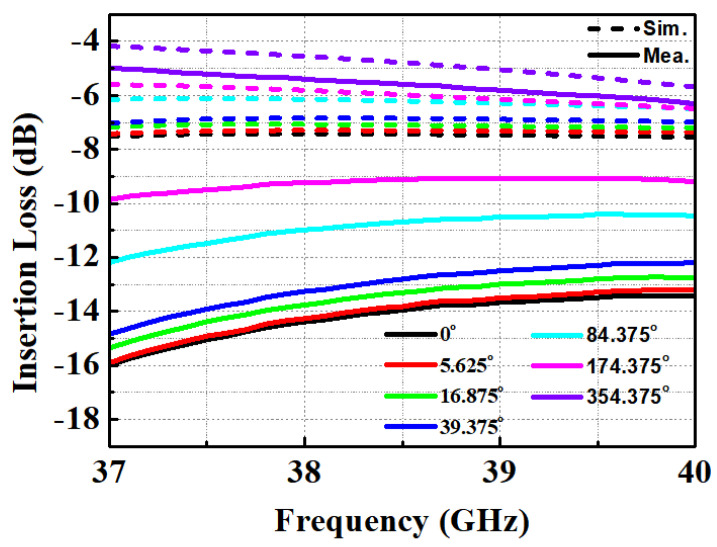
Insertion loss due to delay.

Figure 4 illustrates the input and output return loss (S11 and S22) of the fabricated STPS for various phase steps of each bit. The input return loss (S11) pertains to the input of the 11.25-degree phase shifter, while the output return loss (S22) pertains to the output of the 45-degree phase shifter. These results reveal that the return loss remains better than 10.7 dB across different phase states. Figure 5 displays the insertion loss (S21) of STPS for various phase states, including the insertion loss when delay paths from 0 to 180 degrees are added. Notably, as all phase shifters are transitioned into the delayed state, the insertion loss decreases, approaching a level closely resembling the simulation results. This behavior is primarily attributed to two factors.

Firstly, the significant insertion loss, caused by the 90-degree and 180-degree bit bypass transistors, is a key contributor. This limitation stems from the utilization of smaller transistors in the 90-degree bit bypass circuit. Furthermore, the 180-degree bit comprises two 90-degree bits, amplifying its impact. Secondly, the modeling from the Process Design Kit (PDK) appears to have played a role. It is worth noting that we employed the transistor model provided by the PDK, initially designed up to 26.5 GHz, with discrepancies becoming more pronounced at higher frequencies. Additionally, the capacitors used in the delay circuit were based on the PDK model.

**Figure 6:**
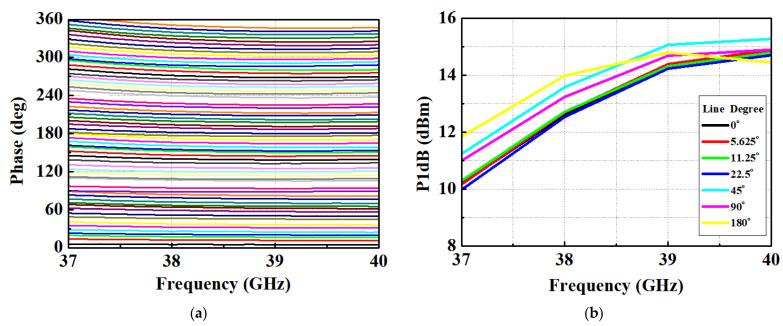
(**a**) Measured phase response; (**b**) Simulated P1dB of n260 phase shifter.

**Figure 7:**
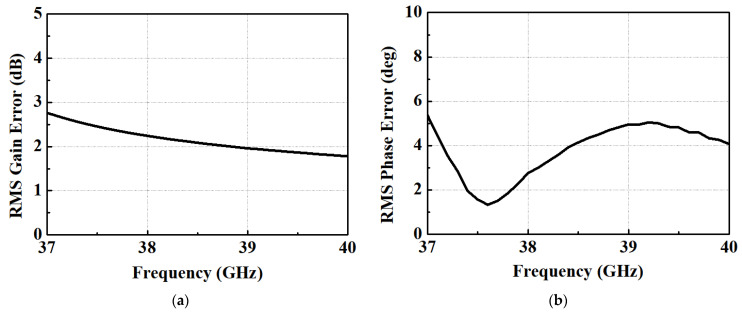
RMS error: (**a**) Gain; (**b**) Phase.

However, the inductor, crucial for generating delay, was reasonably well-simulated through electromagnetic (EM) simulation. Moreover, since the delay circuit was constructed as an LCL network, the influence of the inductor was relatively significant. Consequently, the results obtained for the delay path closely aligned with the simulation. Figure 6a displays the measured phase response of the STPS across 64 phase steps. Figure 6b presents the simulated input 1-dB compression points for various phase steps of each bit. The simulation demonstrates an IP1dB exceeding 10 dBm within the operating frequency band. Notably, a substantial variation in IP1dB is observed in the 37~38 GHz. Figure 7a reveals that the measured and calculated RMS gain error falls within the range of 1.69 to 3.21 dB. Notably, it becomes evident that the variation in insertion loss is more significant at 37 GHz compared to 40 GHz, depending on the phase state. In Figure 7b, it is evident that the RMS phase error for the measured STPS is limited to less than 5.36°. Analyzing Figure 6a, it becomes apparent that each phase aligns after 38 GHz within the operating band. However, a notable observation is the non-constant phase spacing at 37 GHz. Considering the results presented in Figure 5, Figure 6 and Figure 7, it suggests that the fabricated phase shifter shifted to a higher frequency band than initially targeted.

Table 1 presents a performance comparison between the fabricated phase shifter and previous studies. In contrast to other phase shifters, this study achieves a high resolution of 5.625°, good insertion loss per unit bit.

## 4. Conclusions

In this paper, a phase shifter was designed and fabricated to operate within the 5G NR standard n260 frequency range (37~40 GHz) using the 150 nm GaN HEMT process. The fabricated phase shifter is structured as a 6-bit switch filter, demonstrating impressive performance metrics: an RMS phase error of less than 5.36 degrees, an RMS gain error of under 3.21 dB, and a robust IP1dB exceeding 10 dBm. This high IP1dB value proves advantageous for seamless integration with high-power RF circuits.

## Figures and Tables

**Table 1:** Performance Summary and Comparison with the State-of-the-Art Phase Shifters.

	This Work	[11]	[12]	[13]	[14]	[15]	[5]	[19]
Process	150 nm GaN	250 nm BiCMOS	150 nm GaAs	65 nm CMOS	28 nm CMOS	65 nm CMOS	65 nm CMOS	22 nm FD SOI
Topology ***	STPS	APN	STPS	STPS	STPS	STPS	PVSPS	AVSPS
Frequency (GHz)	37~40	37~43	36~39	38~40.5	29~37	35~41.9	32~40	24~36
FBW (%)	7.8	15	8	6.4	24	17.95	22	40
No. of Bit	6	2	4	5	4	5	7	5
Resolution (°)	5.625	45	22.5	11.25	22.5	11.25		
Gain (dB)	−10.5	−13.25	−10.6	−14.5	−12.8	−7.56	−17.5	−6.6
Gain var. (dB)	±5.5	±0.75	±2.9	±2	±2.5	±0.22	±0.6	±1.6
Gain/Bit (dB)	−1.75	−6.63	−2.65	−2.9	3.2	−1.5	−2.5	−1.32
IP1dB (dBm)	>10–16 **	N/A	N/A	N/A	5	>7.1	10.2–13.5	<4
RMS PE (°)	<5.36	<5	<9.7	<4	<8.8	<5.9	<1.6	<4
RMS GE (dB)	<3.21	<0.5	<1.81	<1.4	N/A	<0.22	<0.36	<0.6
Pdc (mW)	0	0	0	0	0	0	0	7.2
Area (mm^2^)	3.36 *	0.28	0.65	0.4	0.08	0.13	0.14	0.58

* Area with including bondpads; ** Simulation result; *** APN: All-Pass Network; STPS: Switched-Type PS; AVSPS: Active Vector Sum PS; PVSPS: Passive Vector Sum PS.

## Data Availability

Not applicable.

## References

[1] Lee J.H., Park J.S., Hong S.C. Frequency Reconfigurable Dual Band CMOS Power Amplifier for Millimeter Wave 5G Communications. Proceedings of the IEEE MTT-S International Microwave Symposium (IMS).

[2] Wang F., Wang H. An Instantaneously Broadband Ultra-Compact Highly Linear PA with Compensated Distributed-Balun Output Network Achieving > 17.8 dBm P1dB and >36.6% PAEP1dB over 24 to 40 GHz and Contunuously Supporting 64-256-QAM 5G NR Signals over 24 to 42 GHz. Proceedings of the 2020 IEEE International Solid-State Circuits Conference—(ISSCC).

[3] Asbeck P., Bharadia D., Galton I., Hall D., Le H.P., Mercier P., Rebeiz G. (2023). Integrated Circuits for Wireless Communications: Research Activities at the University of California, San Diego. IEEE Microwave Magazine.

[4] Anjos E.V.P., Schreurs D.M.M.P., Vandenbosch G.A.E., Geurts M. (2021). A Compact 26.5-29.5-GHz LNA-Phase Shifter Combo with 360° Continuous Phase Tuning Based on All-Pass Networks for Millimeter-Wave 5G. IEEE Trans. Circuits Syst. I.

[5] Li Y., Duan Z., Fang Y., Li X., Deng B., Dai Y., Sun L., Gao H. (2022). A 32-40 GHz 7-bit Bi-Directional Phase Shifter with 0.36 dB/1.6° RMS Magnitude/Phase Errors for Phased Array Systems. IEEE Trans. Circuits Syst. I.

[6] Sellers A.J., Tine C., Kini R.L., Hontz M.R., Khanna R., Lemmon A.N., Shahabi A., New C. Effects of parasitic inductance on performance of 600-V GaN devices. Proceedings of the 2017 IEEE Electric Ship Technologies Symposium (ESTS).

[7] Pace L., Idir N., Thierry D., Jaeger J.C.D. (2021). Parasitic Loop Inductances Reduction in the PCB Layout in GaN-Based Power Converters Using S-Parameters and EM Simulations. Energies.

[8] Pour F.L., Reed R.T., Ha D.S. (2022). Design and performance Investigation of a Temperature Compensated Transmitter with GaN HEMTs for Phased-Array Applications. IEEE Trans. Microw. Theory Tech..

[9] Ross T.N., Hettak K., Cormier G., Wight J.S. (2015). Design of X-Band GaN Phase Shifters. IEEE Trans. Microw. Theory Tech..

[10] He E.T., Zhang Z.H., Xu S. A GaN HEMT 6-Bit Digital Phase Shifter for Millimeter Wave Phased Array System. Proceedings of the 2022 IEEE MTT-S International Microwave Workshop Series on Advanced Materials and Processes for RF and THz Applications (IMWS-AMP).

[11] Anjos E.V.P., Schreurs D.M.M.P., Vandenbosch G.A.E., Geurts M. A 15–43.5 GHz Swiched-Bit Phase Shifter for 5G Mobile Handsets. Proceedings of the 2019 IEEE Asia-Pacific Microwave Conference (APMC).

[12] Yang M.S., Lin Y.T., Kao K.Y., Lin K.Y. A Compact E-Mode GaAs pHEMT Phase Shifter MMIC for 5G Phased-Array Systems. Proceedings of the IEEE Asia-Pacific Microwave Conference.

[13] Tsai J.H., Lin K.J., Xiao H. A 39 GHz 5-Bit Switch Type Phase Shifter using 65 nm CMOS Technology. Proceedings of the 2019 IEEE 8th Global Conference on Consumer Electronics (GCCE).

[14] Jung M., Min B.W. (2020). A compact Ka-band 4-bit phase shifter with low group delay deviation. IEEE Microw. Wirel. Compon. Lett..

[15] Lin Y.H., Tsai Z.M. (2021). A Wideband Compact 5-Bit Phase Shifter with Low Loss and RMS Errors for 5G Applications. IEEE Microw. Wirel. Compon. Lett..

[16] Meng F., Ma K., Yeo K.S., Xu S. (2016). A 57-to-64-GHz 0.094-mm² 5-bit passive phase shifter in 65-nm CMOS. IEEE Trans. Very Large Scale Integr. (VLSI) Syst..

[17] Xia J., Farouk M., Boumaiza S. Digitally-assisted 27-33 GHz reflection-type phase shifter with enhanced accuracy and low IL-variation. Proceedings of the 2019 IEEE Radio Frequency Integrated Circuits Symposium (RFIC).

[18] Kadam M., Kumar A., Aniruddhan S. (2020). A 28 GHz reflective-type transmission-line-based phase shifter. IEEE Trans. Circuits Syst. I..

[19] Bardeh M.G., Fu J., Naseh N., Paramesh J., Entesari K. (2023). A Wideband Low RMS Phase/Gain Error mm-Wave Phase Shifter in 22-nm CMOS FDSOI. IEEE Microw. Wireless Technol. Lett..

[20] Wang S., Park J., Hong S. (2021). A K-bnad variable-gain phase shifter based on Gilbert-cell vector synthesizer with RC-RL poly-phase filter. IEEE Microw. Wirel. Compon. Lett..

[21] Park J., Jeong G., Hong S. (2020). A Ka-band variable-gain phase shifter with multiple vector generators. IEEE Trans. Circuits Syst. II Exp. Briefs..

[22] Kodak U., Rebeiz G.M. (2019). A 5G 28-GHz common-leg T/R front-end in 45-nm CMOS SOI with 3.7-dB NF and -30-dBc EVM with 64-QAM/500-MBaud modulation. IEEE Trans. Microw. Theory Tech..

[23] Eblabla A., Li X., Wallis D.J., Guiney I., Elgaid K. (2018). High-Performance MMIC Inductors for GaN-on-Low-Resistivity Silicon for Microwave Applications. IEEE Microw. Wirel. Compon. Lett..

[24] Chander S., Bansal K., Gupta S., Gupta M. Design and Analysis of High Performance Air-Bridge spiral circular inductors for GaN MMICs up to Ku Band. Proceedings of the 2017 Devices for Integrated Circuit (DevIC).

[25] Burghartz J.N., Edelstein D.C., Soyuer M., Ainspan H.A., Jenkins K.A. (1998). RF Circuit Design Aspects of Spiral Inductors on Silicon. IEEE J. Silid-State Circuits.

[26] Lee H.S., Min B.W. (2015). W-Band CMOS 4-bit Phase Shifter for High Power and Phase Compression points. IEEE Trans. Circuits Syst. II Exp. Briefs.

